# Development and validation of a non-invasive model for predicting significant fibrosis based on patients with nonalcoholic fatty liver disease in the United States

**DOI:** 10.3389/fendo.2023.1207365

**Published:** 2023-09-05

**Authors:** Yuanhui Guo, Baixuan Shen, Yanli Xue, Ying Li

**Affiliations:** ^1^ Endocrine and Metabolic Disease Center, Medical Key Laboratory of Hereditary Rare Diseases of Henan, Luoyang Sub-center of National Clinical Research Center for Metabolic Diseases, First Affiliated Hospital, College of Clinical Medicine of Henan University of Science and Technology, Luoyang, China; ^2^ Department of Pharmacy, First Affiliated Hospital of Henan University of Science and Technology, College of Clinical Medicine of Henan University of Science and Technology, Luoyang, China

**Keywords:** non-alcoholic fatty liver disease, liver fibrosis, prediction model, logistic regression, NHANES

## Abstract

**Background:**

Liver fibrosis is closely related to abnormal liver function and liver cancer. Accurate noninvasive assessment of liver fibrosis is of great significance for preventing disease progression and treatment decisions. The purpose of this study was to develop and validate a non-invasive predictive model for the asses`sment of significant fibrosis in patients with non-alcoholic fatty liver disease.

**Methods:**

Information on all participants for 2017-2018 was extracted from the NHANES database. The eligible patients with significant fibrosis (n=123) and non-significant fibrosis (n=898) were selected to form the original dataset. Variable selection was performed using least absolute shrinkage and selection operator (Lasso) regression, and multivariate logistic regression analysis was used to develop a prediction model. The utility of the model is assessed in terms of its discrimination, calibration and clinical usability. Bootstrap-resampling internal validation was used to measure the accuracy of the prediction model.

**Results:**

This study established a new model consisting of 9 common clinical indicators and developed an online calculator to show the model. Compared with the previously proposed liver fibrosis scoring system, this model showed the best discrimination and predictive performance in the training cohort (0.812,95%CI 0.769-0.855) and the validation cohort (0.805,95%CI 0.762-0.847), with the highest area under curve. Specificity(0.823), sensitivity(0.699), positive likelihood ratio(3.949) and negative likelihood ratio(0.366) were equally excellent. The calibration plot of the predicted probability and the actual occurrence probability of significant fibrosis shows excellent consistency, indicating that the model calibration is outstanding. Combined with decision curve analysis, this model has a great benefit in the range of 0.1-0.8 threshold probability, and has a good application value for the diagnosis of clinical significant fibrosis.

**Conclusion:**

This study proposes a new non-invasive diagnostic model that combines clinical indicators to provide an accurate and convenient individualized diagnosis of significant fibrosis in patients with non-alcoholic fatty liver disease.

## 1 Introduction

Non-alcoholic fatty liver disease (NAFLD) is the most common liver disease worldwide and may progress to liver fibrosis, cirrhosis and hepatocellular carcinoma, with a global prevalence of approximately 25% in the general population (1). NAFLD is strongly associated with features of the metabolic syndrome, including insulin resistance and obesity, and it has become a major cause of the global increase in chronic liver disease and will continue to grow exponentially in the future, posing a huge challenge to global public health systems (2, 3). NAFLD is defined as the accumulation of fat in the liver (>5%) after excluding underlying factors such as viral infections, drugs, alcohol, etc. Non-alcoholic steatohepatitis (NASH) is defined as the presence of hepatocellular damage and cell death with lobular and portal inflammation and is the next entity in the spectrum of the disease, culminating in the final stages of fibrosis and cirrhosis in the presence of collagen deposition and vascular remodelling (4, 5). The disease has a range of histological features, from steatosis without fibrosis to NASH with various stages of fibrosis (6). The Metavir scoring system is widely used for the assessment of liver fibrosis (7) and the staging is defined as follows: F0:no fibrosis; F1:portal fibrosis without septa; F2:portal fibrosis with a few septa extending beyond the portal vein; F3:bridging fibrosis or a large number of septa without cirrhosis; F4:cirrhosis. Notably, liver fibrosis is a substantial predictor of relevant clinical events, both in terms of overall mortality and liver-related morbidity and mortality (8, 9). It is therefore a great challenge to accurately identify NAFLD patients with pathologically important in a way that is non-invasive and affordable to the healthcare system.

The current methods are mainly divided into two categories: serum biomarkers obtained by laboratory examination or imaging examination. In past studies, several serological models have been developed for the prediction of liver fibrosis based on biochemical markers and clinical information (10–12). Although serological markers can provide dynamic information on fibrosis progression, there is no single non-invasive serological marker that can accurately predict liver fibrosis progression (13). In recent years, scoring systems based on the joint development of several serological indicators have been proposed, including the aspartate aminotransferase-to-alanine aminotransferase ratio (AST/ALT) (14), the Forns Index (15), the fibrosis-4 index (FIB-4) (16), BARD score (17), and aspartate aminotransferase-to-platelet ratio index (APRI) (18), which are widely used to assess the progression of liver fibrosis, where the required indicators can be calculated from clinical features and routine biochemical tests. However, when these scoring systems are used to predict fibrosis progression in patients with NAFLD, they do not appear to perform well, as most of these models were developed based on populations with chronic liver disease, such as viral hepatitis. Transient elastography (TE) is an ultrasound-based non-invasive method that uses shear wave velocity to provide a measure of liver stiffness and a controlled attenuation parameter (CAP) for the assessment of liver fibrosis and steatosis. Compared to a liver biopsy, TE has a larger measurement area and is 100 times larger than the volume of tissue obtained from a biopsy (19). A 2016 head-to-head comparison of nine fibrosis tests identified TE as the most accurate method for the non-invasive diagnosis of fibrosis in patients with NAFLD (20). In addition to high accuracy, the meta-analysis demonstrated that TE results have remarkable prognostic value (21). Transient elastography has been approved by the Food and Drug Administration (FDA) as a test for the assessment of liver fibrosis, but its application is limited by the condition of the equipment and the requirements of specialist technicians (22, 23).

The aim of this study was to establish a prediction model for significant liver fibrosis based on biochemical indexes and clinical features of NAFLD patients, and to develop a web calculator belonging to this model for directly calculating the probability of fibrosis occurrence, which greatly enhances the efficiency of using the model. The establishment of this model is expected to provide great convenience for the diagnosis of significant hepatic fibrosis in NAFLD patients, thus enhancing the efficiency of frontline clinicians.

## 2 Methods

### 2.1 Study design and data source

The National Health and Nutrition Examination Survey (NHANES) is a multi-year, cross-sectional, nationally representative survey of the U.S. population designed to assess the health and nutritional status of a representative sample of U.S. residents and NHANES survey data is fully open to researchers. The survey, conducted by the National Center for Health Statistics (NCHS), followed a complex, stratified, multi-stage probabilistic design that included dietary, examination, laboratory and questionnaire, with data collected every two years (24). The NCHS Research Ethics Review Committee approved the NHANES investigation protocol and informed consent was provided to all participants, thus allowing our study to be granted an exemption from ethical review. The target population of NHANES is the non-institutionalized civilian resident population of the United States. The design of NHANES changes periodically to sample more certain subgroups of specific public health interest to improve the reliability and accuracy of estimates of health status indicators for these population subgroups. NHANES uses a complex multi-stage probability design to sample the non-institutionalized population residing in the 50 states and Washington, DC. We conducted a cross-sectional study using NHANES data (n=9254) for the period 2017-2018.

According to the latest update of the European Association for the Study of the Liver (EASL) clinical practice guidelines on the use of non-invasive tests for the assessment of liver disease, participants with a CAP score above 275 dB/m were diagnosed with hepatic steatosis (25). A large meta-analysis based on assessing diagnostic thresholds for CAP in NAFLD defined a CAP score ≥ 248 dB/m as NAFLD (26) and participants with CAP < 248 dB/m were considered non-NAFLD and excluded. Of the 9254 participants included in the study, 5494 completed elastography(fasting time of at least 3 hours, 10 or more complete stiffness measures, and a liver stiffness interquartile range/median stiffness<30%). 493 participants completed part of the examination(either a fasting time<3 hours, <10 complete stiffness measures, or a liver stiffness interquartile range/median stiffness 30% or higher), 258 participants were ineligible(see eligibility criteria above), and 156 participants were not done(refusal, limited time during exam visit, other), they were all excluded from this study. This data was provided by the LUAXSTA file. Participants with hepatocellular carcinoma, autoimmune hepatitis, hepatitis B, and hepatitis C were also excluded from this study, and data were obtained from MCQ230A-C, MCQ510E, LBDHBG, LBDHCI, and LBXHCR file. Participants judged to be excessive drinkers were similarly excluded due to the strong association between excessive drinking and chronic liver disease. Excessive drinking was defined as mean alcohol consumption >20g/day for men and > 10g/day for women (27). Alcohol consumption data were obtained from DR1TALCO and DR2TALCO files, representing the daily alcohol consumption of participants in the two 24-hour reviews. If participants completed two 24-hour reviews, we used the mean of the two drinking sessions as the mean alcohol intake, otherwise, only data from the first 24-hour review were used. Finally missing values in the remaining variables were removed and only the complete data were included in the analysis, with a total of 1021 patients with NAFLD who met the inclusion criteria eligible for the follow-up analysis.

### 2.2 Outcome

The outcome of this study was significant fibrosis, defined as F2-F4 using the Brunt & Kleiner, Metavir, Ludwig, or SAF scoring system. A cross-sectional, prospective multicentre study following the Standards for Reporting of Diagnostic Accuracy (STARD) defined median liver stiffness≥8.2 kPa as Significant fibrosis (22). The degree of liver fibrosis is measured by the FibroScan, which uses ultrasound and vibration-controlled transient elastography to derive liver stiffness. All participants aged 12 years and above are eligible to participate. Participants were excluded if they (a) were unable to lie on the examination bed, (b) were pregnant (or unsure if they were pregnant) at the time of the examination, or were unable to obtain urine for pregnancy testing, (c) had an electronic medical device implanted, or (d) were wearing a bandage or had lesions in the right ribs of the abdomen (where the measurement would be taken). The elastography measurements were obtained in the NHANES Mobile Examination Center (MEC), using the FibroScan model 502 V2 Touch equipped with a medium (M) or extra large (XL) wand (probe). With FibroScan, a mechanical vibration of mild amplitude and low frequency (50Hz) is transmitted through the intercostal space using a vibrating tip contacting the skin. The vibration induces a shear wave that propagates through the liver. The displacements induced by the shear waves are tracked and measured using pulse echo ultrasound acquisition algorithms. The shear wave velocity is related directly to tissue stiffness; with harder tissues, there is faster shear wave propagation. Using the Young modulus, the velocity is converted into liver stiffness, and expressed in kilopascals. The LUXSMED file provides information on median liver stiffness.

### 2.3 Predictor variables

Patient demographic data, biochemical indicators, and clinical characteristics were extracted as candidate predictors to be used in building the multifactorial prediction model. Smoking data were from SMQ020 and SMQ040, and the file meanings were ‘Smoked at least 100 cigarettes in life’ and ‘Do you now cigarette smokes?’. Smoke (Yes) was defined as SMQ020 answering ‘Yes’ while SMQ040 answered ‘Every day’ or ‘Some days’ or ‘Not at all’, otherwise, Smoke was defined as No. We obtained the hypertension data from the BPQ020 file in the Questionnaire project. The meaning of the BPQ020 file is ‘Ever told you had high blood pressure’, as long as the patient answered ‘Yes’ to this item, they are defined as hypertensive. Diabetes is a common clinical disease, and we have adopted multiple indicators to define it. Participants with diabetes were defined as having any one of the following: (a) hemoglobin A1C concentration≥ 6.5% or a fasting plasma glucose level ≥ 126 mg/dL (28); (b) for those who responded ‘yes’ to the question: ‘Doctor told you have diabetes?’ or ‘Taking insulin now?’. The LBXGH, LBXGLU, DIQ010, and DIQ050 files provide the relevant information. Age, Sex, BMI (Body Mass Index), ALT (Alanine aminotransferase), AST (Aspartate aminotransferase), ALP (Alkaline phosphatase), GGT (γ-glutamyl transpeptidase), Platelet count, Hemoglobin, Glycosylated hemoglobin, Glucose, Insulin, Albumin, Ferritin, Triglyceride, Total bilirubin, Total cholesterol, LDL (Low-density lipoprotein), HDL (High-density lipoprotein) were also included as candidate predictors. RIDAGEYR, RIAGENDR, BMXBMI, LBXSATSI, LBXSASSI, LBXSAPSI, LBXSGTSI, LBXPLTSI, LBXHGB, LBXGH, LBDSGLSI, LBXIN, LBDSALSI, LBDFERSI, LBDSTRSI LBDSTBSI, LBDSCHSI, LBDLDMSI, LBDHDDSI are the variable codes for the above candidate predictors in the NHANES database, which provide specific information on variable descriptions, laboratory methodological descriptions, laboratory method documentation, laboratory quality assurance and testing, data processing and editing to ensure that all variables are measured scientifically and accurately. In this study, the relationship between predictors and outcomes was double-blinded.

### 2.4 The existing noninvasive liver fibrosis scoring system

AST/ALT, Forns index, FIB-4 index, BARD scoring system, and APRI indicators are considered to be valid for non-invasive assessment of liver fibrosis. Forns Index =7.811–3.131× ln (platelet count(×10^9^/L))+0.781×ln(GGT(U/L))+3.467×ln(age(years))−0.014×ln(cholesterol (mg/dL)) (15), FIB-4=age(years)×AST(U/L)/[ALT(U/L)1/2×platelet count (×10^9^/L)] (16), BARD=BMI≥28(Yes=1,No=0)+AST/ALT≥0.8(Yes=2,No=0)+ diabetes (Yes=1,No=0) (17), APRI =AST(U/L)/upper limit of normal (set at 40U/L)×100/platelet count (×10^9^/L) (18).

### 2.5 Data processing

The predictor variables were treated as follows to make them normally distributed and better linearly related to the outcome: (a) continuous variables with skewness distribution were log-transformed to make them normally distributed. (b) Restricted cubic spline (RCS) was used to test the linear relationship between continuous variables and outcome. The continuous variables without or with poor linear relationship were logarithmically, exponentially or squarely converted to fit the linear relationship between variables and outcome.

### 2.6 Variable screening and model establishment

**Figure 1:**
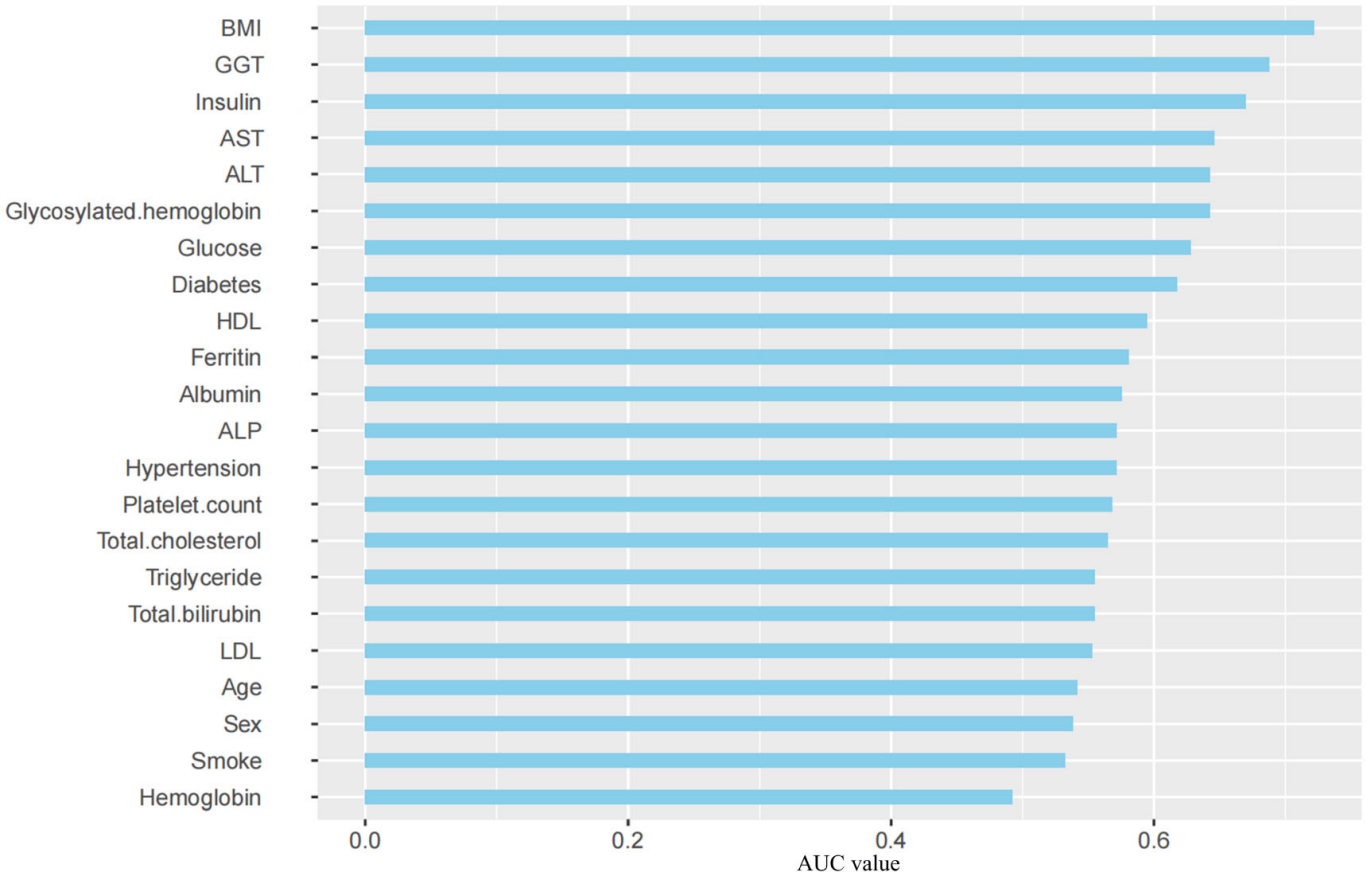
AUC Distribution of Candidate Predictors. BMI, Body Mass Index; GGT, γ-glutamyl transpeptidase; AST, Aspartate aminotransferase; ALT, Alanine aminotransferase; HDL, High-density lipoprotein; ALP, Alkaline phosphatase; LDL, Low-density lipoprotein; AUC, Area Under Curve.

**Figure 2:**
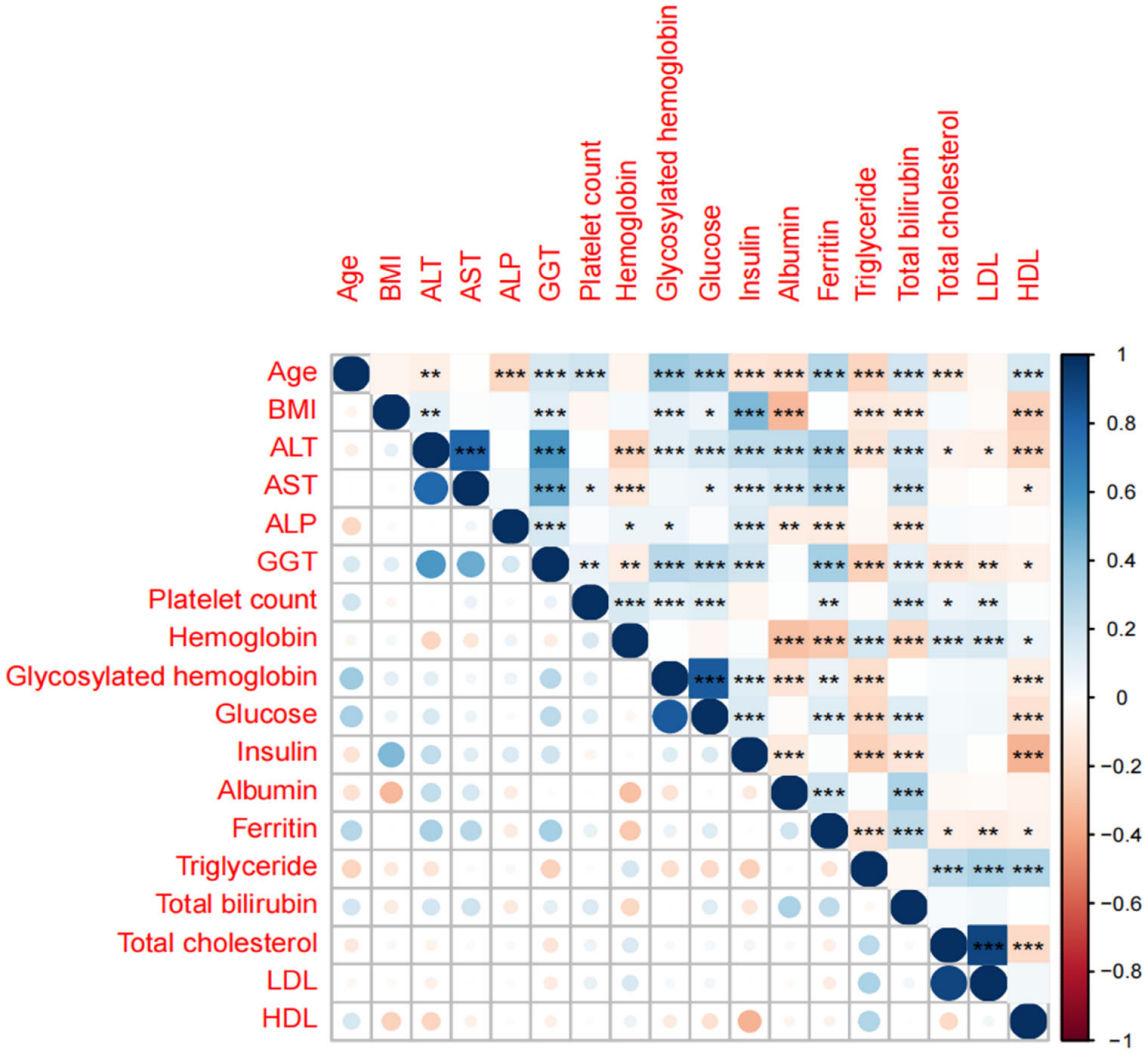
Correlation analysis heat map of candidate predictors. *, **, *** is significant correlation markers, which represent the degree of correlation between two variables.

A single-factor analysis was conducted to calculate the area under curve (AUC) values for each candidate predictor and to plot the AUC bars in a longitudinal decreasing order (**Figure 1**). In addition, to present the correlation between all candidate predictors, a correlation heat map (**Figure 2**) was drawn including all continuous predictors, and the degree of correlation between candidate predictors was labeled in the figure. Combined with the analysis of the above results, the AUC values of all the variables collected, except for the variable Hemoglobin, were greater than 0.5, while the degree of correlation between the variables was within acceptable limits, so we performed a multifactorial analysis to select the final predictors.

In order to determine a reliable set of predictors, we use the Lasso method (29), by setting the penalty coefficient λ, select the variables with good correlation with the outcome and the regression coefficient β≠0 from the alternative predictors as the predictors of the final model and use multivariate logistic regression analysis to calculate the specific parameters of each predictor to predict possible diagnosis. In order to give front-line clinicians a convenient and practical diagnostic tool for liver fibrosis, this study established a prediction model based on multivariate logistic regression analysis and constructed a liver fibrosis probability calculator.

### 2.7 Model validation

We performed bootstrap-resampling internal validation to measure the accuracy of the prediction model. The internal verification follows the following steps: (a) Using the put-back sampling, a resampling data set with the same sample size as the model development queue is reconstructed as the training set; (b) Implement a complete model training process in the training set and calculate the model performance in the training set; (c) In the original model development queue, the model performance of the above model is calculated, and the difference between the two model performances is calculated as the overestimation (optimism); (d) Repeat the above process 100 times to obtain the high estimates of 100 models; (e) Calculate the mean value of the high valuation of 100 models as the high valuation adjustment; (f) The performance of the model in the original data minus the high valuation adjustment as the model performance in the internal verification. Compared with the traditional random split verification, bootstrap resampling verification has higher utilization efficiency of data and avoids the problem of small verification sample size.

### 2.8 Evaluation method of model prediction effect

The receiver operating characteristic curve (ROC) of the model was drawn and the AUC and its 95% confidence interval (CI) were calculated to evaluate the discrimination of the model. The calibration curve is used to evaluate the calibration of the model. Model discrimination refers to the ability of the model to correctly distinguish between high-risk and low-risk individuals, that is, the ability of the model to correctly classify whether the outcome event occurs, which is usually evaluated by AUC. The prediction effect of the model with an AUC range of 0.5-0.7 is considered as poor, 0.7-0.8 as general, 0.8-0.9 as good, and 0.9 or more as excellent (30). Model calibration can evaluate whether the absolute probability (absolute risk) of model prediction is accurate. Decision curve analysis (DCA) is introduced to visually display the net income under different threshold probabilities to reflect the clinical utility of the model. The confusion matrix was used to calculate the model specificity, sensitivity, Youden ‘s index, positive predictive value, negative predictive value, positive likelihood ratio, and negative likelihood ratio further reflected the model performance.

## 3 Result

### 3.1 Study population

**Figure 3:**
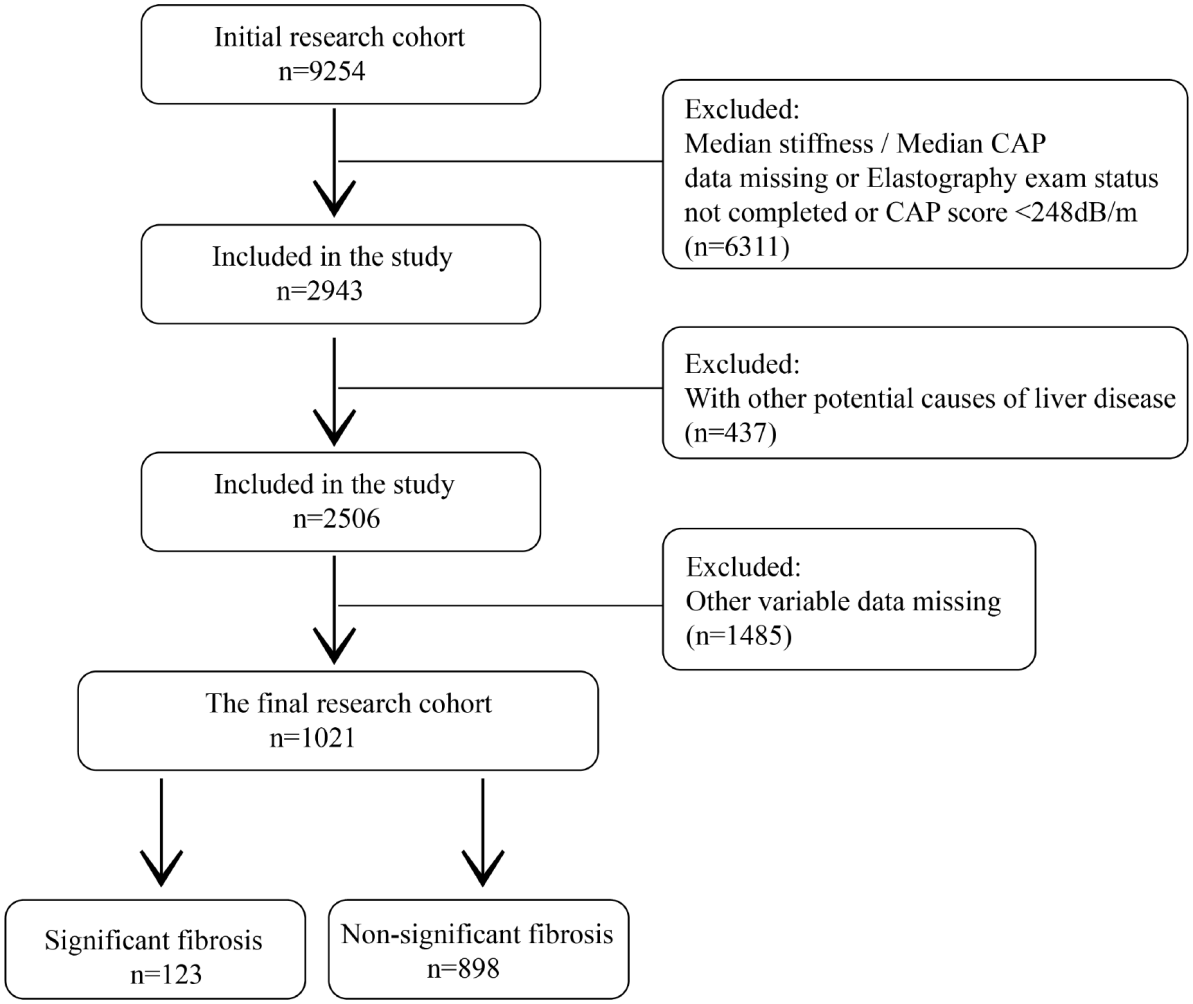
Flow chart of this study. CAP, Controlled attenuation parameter.

Among the 9254 people initially included in the study, 6311 non-NAFLD patients who did not complete elastography and liver stiffness measurements, and CAP data were missing were excluded. At the same time, patients with potential causes of chronic liver disease (437) and patients with missing values of other variables (1485) were also excluded. A total of 1021 patients were included in the study, including 123 significant fibrosis and 898 non-significant fibrosis (**Figure 3**).

In the original data set, there were 504 males (49.4%) and 517 females (50.6%). The ratio of male to female was 0.97: 1, and the median age of the whole cohort was 54. All continuous variables (Age, BMI, ALT, AST,ALP, Platelet count, GGT, Hemoglobin, Glycosylated hemoglobin, Glucose, Insulin, Albumin, Ferritin, Triglyceride, Total bilirubin, Total cholesterol, LDL, HDL) in the cohort were expressed as median (interquartile range). The categorical variables (Sex, Smoke, Hypertension, Diabetes) show the percentage of each category in the total. The subjects were grouped according to whether significant fibrosis occurred. The baseline characteristics of the two groups were compared. The statistical test p values of continuous variables and categorical variables were calculated by Mann-Whitney test and chi-square test, respectively. P<0.05 was statistically significant (**Table 1**).

**Table 1:** Patient characteristics of the model development cohort.

Characteristics	Overall	Significant fibrosis=No	Significant fibrosis=Yes	P value
	n=1021	n=898	n=123	
Age (years)	54.00 [35.00,65.00]	54.00 [35.00, 64.00]	57.00 [41.50, 66.50]	0.135
Sex = male (%)	504 (49.4)	435 (48.4)	69 (56.1)	0.134
BMI (kg/m^2^)	31.20 [27.30,35.80]	30.70 [27.00, 35.10]	35.80 [31.90, 42.75]	<0.001
ALT (U/L)	19.00 [14.00,28.00]	19.00 [14.00, 27.00]	25.00 [17.00, 45.50]	<0.001
AST (U/L)	19.00 [16.00,24.00]	19.00 [15.25, 23.00]	22.00 [18.00, 34.00]	<0.001
ALP (IU/L)	80.00 [67.00,97.00]	80.00 [66.00, 96.75]	85.00 [70.50, 108.00]	0.01
GGT (IU/L)	22.00 [16.00,33.00]	22.00 [15.00, 31.00]	32.00 [22.00, 56.00]	<0.001
Platelet count (1000 cells/uL)	241.00 [200.00,285.00]	242.00 [204.00,286.00]	225.00 [179.50, 278.00]	0.004
Hemoglobin (g/dL)	14.10 [13.20,15.20]	14.10 [13.20, 15.10]	14.30 [13.35, 15.60]	0.08
Glycosylated hemoglobin (%)	5.70 [5.40, 6.20]	5.70 [5.40, 6.10]	6.10 [5.60, 7.35]	<0.001
Glucose (mmol/L)	5.55 [5.11, 6.33]	5.50 [5.11, 6.27]	6.00 [5.36, 7.58]	<0.001
Insulin (uU/mL)	13.67 [9.20, 22.08]	13.15 [8.85, 20.52]	21.43 [12.31, 30.90]	<0.001
Albumin (g/L)	40.00 [38.00,42.00]	40.00 [38.00, 42.00]	39.00 [37.00, 41.00]	0.007
Ferritin (ug/L)	112.00 [50.80,203.00]	108.00 [49.02, 196.75]	142.00 [67.50, 246.50]	0.004
Triglyceride (mmol/L)	1.39 [1.00, 1.92]	1.37 [0.98, 1.92]	1.50 [1.10, 1.96]	0.102
Total bilirubin (umol/L)	6.84 [5.13, 10.26]	6.84 [5.13, 10.26]	8.55 [5.13, 11.97]	0.047
Total cholesterol (mmol/L)	4.66 [4.03, 5.38]	4.69 [4.06, 5.43]	4.45 [3.88, 5.19]	0.02
LDL (mmol/L)	2.82 [2.28, 3.46]	2.85 [2.30, 3.49]	2.71 [2.08, 3.31]	0.058
HDL (mmol/L)	1.22 [1.06, 1.42]	1.24 [1.06, 1.42]	1.11 [1.01, 1.29]	0.001
Smoke = Yes (%)	391 (38.3)	337 (37.5)	54 (43.9)	0.206
Hypertension = Yes (%)	437 (42.8)	369 (41.1)	68 (55.3)	0.004
Diabetes = Yes (%)	312 (30.6)	249 (27.7)	63 (51.2)	<0.001
Significant.fibrosis =Yes (%)	123 (12.0)	0 (0.0)	123 (100.0)	<0.001

BMI, Body Mass Index; GGT, γ-glutamyl transpeptidase; AST, Aspartate aminotransferase; ALT, Alanine aminotransferase; HDL, High-density lipoprotein; ALP, Alkaline phosphatase; LDL, Low-density lipoprotein.

### 3.2 Final predictor variables

**Figure 4:**
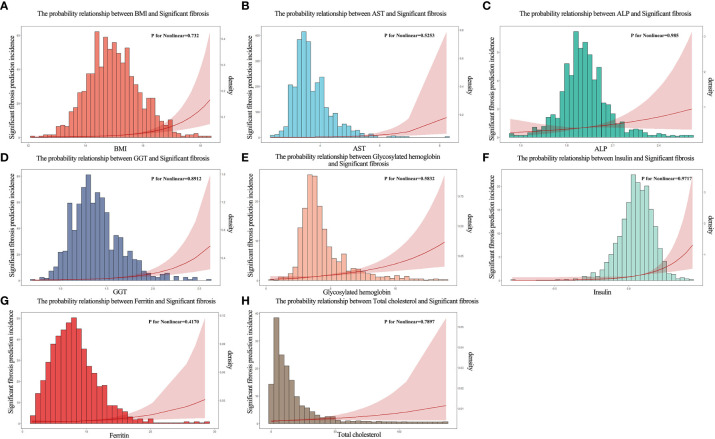
The probability density histogram combined with restricted cubic spline diagram of the final predictors. **(A)** The probability relationship between BMl and significant fibrosis. **(B)** The probability relationship between AST and significant fibrosis. **(C)** The probability relationship between ALP and significant fibrosis. **(D)** The probability relationship between GGT and significant fibrosis. **(E)** The probability relationship between glycosylated hemoglobin and significant fibrosis. **(F)** The probability relationship between insulin and significant fibrosis. **(G)** The probability relationship between ferritin and significant fibrosis. **(H)** The probability relationship between total cholesterol and significant fibrosis.

Based on the literature review of the research preparation phase, we extracted 22 potential variables from the NHANES database as candidate predictors of significant fibrosis outcomes. First, the AUC values of all candidate predictors were calculated and correlation analysis was performed. Subsequently, the lasso method was used to select 9 parameters with non-zero coefficients from all candidate predictors as the final predictors, and the regression coefficients β, standard error, variance inflation factor (VIF), odds ratio (OR) and its 95%CI and p-value (**Table 2**) of each predictor were calculated by multivariate logistic regression analysis. Multicollinearity refers to the linear correlation between independent variables. The greater the degree of multicollinearity, the greater the impact on the variance analysis results of the model and the prediction effect of the model to a certain extent. In this paper, the colinearity of each predictor is screened, and VIF is used to evaluate the severity of multicollinearity. It is generally believed that VIF is meaningful between 1 and 10, and the closer VIF is to 1, the lighter the degree of multicollinearity. It was found that the VIF values were between 1 and 2, indicating that the selected variables met the requirements. In addition, the P value of the nonlinear relationship between the predictor and the outcome was calculated by variance analysis. The results showed that the nonlinear relationship p-value (P for Nonlinear) of all variables was>0.05, that is, there was a good linear relationship between these variables and the outcome. We draw a bi-coordinate diagram of probability density histogram combined with RCS, which fully demonstrates the distribution of continuous predictors and further visualizes the linear relationship between them and the outcome (**Figure 4**).

**Table 2:** Predictors of significant fibrosis outcome.

Variable	β	Standard error	VIF	OR (95%CI)	p-value
BMI	0.86	0.13	1.16	2.37(1.85, 3.08)	<0.001
AST	0.52	0.17	1.42	1.69(1.20, 2.37)	0.002
ALP	0.78	0.77	1.10	2.17(0.47, 9.80)	0.3
GGT	1.43	0.48	1.61	4.16(1.62, 10.7)	0.003
Glycosylated hemoglobin	0.30	0.17	1.99	1.35(0.96, 1.91)	0.083
Insulin	0.86	1.08	1.17	2.37(0.29, 20.4)	0.4
Ferritin	0.05	0.03	1.17	1.05(0.99, 1.12)	0.12
Total cholesterol	0.02	0.01	1.08	1.02(1.01, 1.03)	0.002
Diabetes	0.27	0.30	1.99	NA	NA
No	NA	NA	NA	NA	NA
Yes	NA	NA	NA	1.31(0.72, 2.37)	0.4

β, regression coefficient; VIF, variance inflation factor; OR, odds ratio; CI, confidence interval; BMI, Body Mass Index; AST, Aspartate aminotransferase; ALP, Alkaline phosphatase; GGT, γ-glutamyltranspeptidase; NA, not applicable.

### 3.3 Model effectiveness comparison and validation

**Figure 5:**
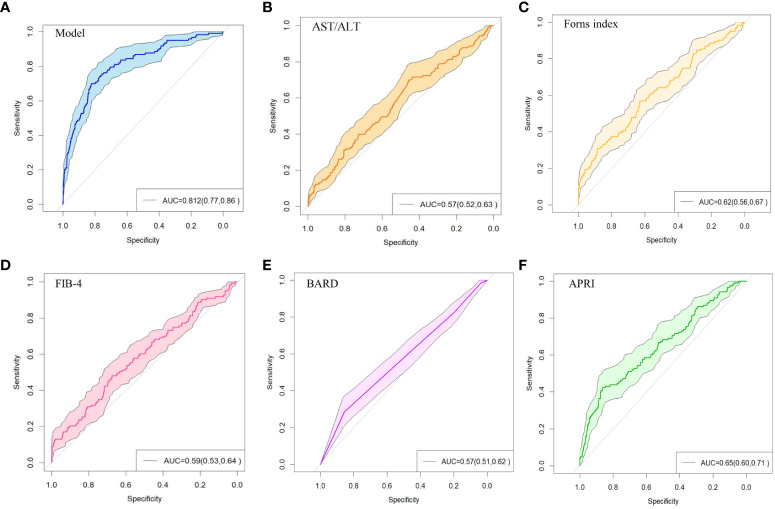
ROC curve of non-invasive liver fibrosis prediction models. **(A)** ROC curve of the model constructed in this paper in the original dataset. **(B)** ROC curve of the AST/ALT model in the original dataset. **(C)** ROC curve of the Forns Index model in the original data set. **(D)** ROC curve of FIB-4 model in the original data set. **(E)** ROC curve of BARD model in the original dataset. **(F)** ROC curve of APRI model in the original dataset.

**Figure 6:**
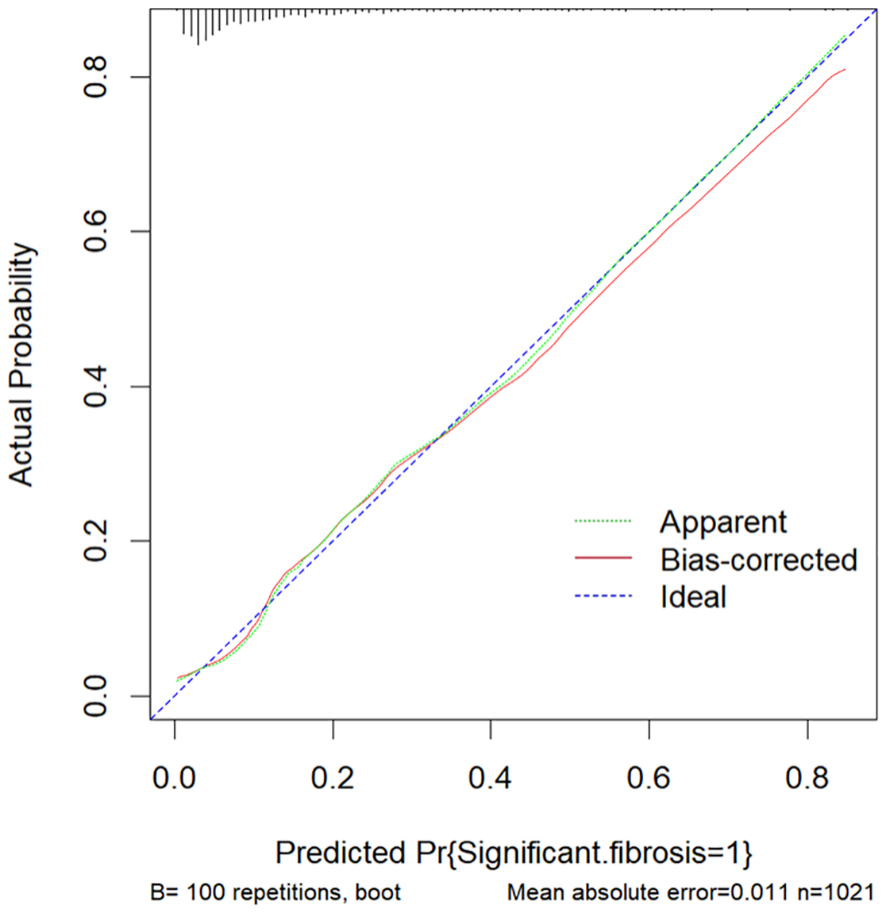
The calibration curve of this model. The Ideal line represents the perfect prediction of the ideal model. The apparent line represents the training performance of the model, while the Bais-corrected line represents the model performance after bootstrap-sampling internal validation, which corrects the overfitting.

The above nine predictors were included in multivariate logistic regression to construct a prediction model. At the same time, the performance of other non-invasive liver fibrosis prediction models was calculated and compared based on the original data set. The ROC curves of all the above models in the original dataset were drawn and the AUC (95% CI) was marked (**Figure 5**). The AUC value of this model is 0.812, which is the highest among all models, reflecting its best discrimination. After bootstrap internal verification, the model can still obtain higher and less variable AUC values, showing the superior accuracy and stability of the model. The effect evaluation indicators of each model in the original data set and bootstrap internal validation data set are listed in detail (**Table 3**). This model has the highest Youden ‘s index, which indicates the total ability of the diagnostic test to find real patients and non-patients. The greater the value, the higher the accuracy of the diagnostic test, and is not affected by the prevalence. Likelihood ratio (LR) refers to the ratio of the probability of a certain test result (such as positive or negative) in a patient in a diagnostic test to the probability of a corresponding result in a non-patient. It is a composite indicator that reflects both sensitivity and specificity. The models performed equally well on the LR metrics in both the original dataset and the bootstrap internal validation dataset. The specificity, sensitivity, positive predictive value, and negative predictive value metrics for each model are also recorded in the table. The calibration curves show that the actual observed outcome incidence did not deviate significantly from the predicted outcome incidence, indicating that the model was well-calibrated (**Figure 6**).

**Table 3:** Efficacy of non-invasive models for the diagnosis of significant fibrosis in patients with nonalcoholic fatty liver disease.

Method	AUC	95%CI	Sp	Se	Youden’s index	NPV	PPV	LR^+^	LR^−^
Original cohort									
Model	0.812	[0.769-0.855]	0.823	0.699	0.522	0.952	0.351	3.949	0.366
AST/ALT	0.573	[0.518-0.629]	0.455	0.699	0.154	0.917	0.150	1.283	0.662
Forns index	0.618	[0.562-0.674]	0.629	0.569	0.198	0.914	0.174	1.534	0.685
FIB-4	0.587	[0.531-0.642]	0.675	0.480	0.155	0.904	0.168	1.477	0.770
BARD	0.569	[0.513-0.624]	0.856	0.285	0.141	0.897	0.213	1.979	0.835
APRI	0.651	[0.596-0.706]	0.864	0.423	0.287	0.916	0.299	3.110	0.668
Bootstrap cohort									
Model	0.805	[0.762-0.847]	0.680	0.805	0.485	0.962	0.256	2.516	0.288
AST/ALT	0.555	[0.503-0.608]	0.339	0.805	0.144	0.927	0.143	1.218	0.575
Forns index	0.579	[0.526-0.633]	0.530	0.634	0.164	0.914	0.156	1.349	0.691
FIB-4	0.560	[0.505-0.615]	0.900	0.211	0.111	0.893	0.224	2.110	0.877
BARD	0.558	[0.509-0.608]	0.470	0.634	0.104	0.904	0.141	1.196	0.779
APRI	0.557	[0.504-0.610]	0.428	0.691	0.119	0.910	0.142	1.208	0.722

Sp, Specificity; Se, Sensitivity; PPV, positive predictive value; NPV, negative predictive value; LR^+^, positive likelihood ratio; LR^-^, negative likelihood ratio.

### 3.4 Clinical use

**Figure 7:**
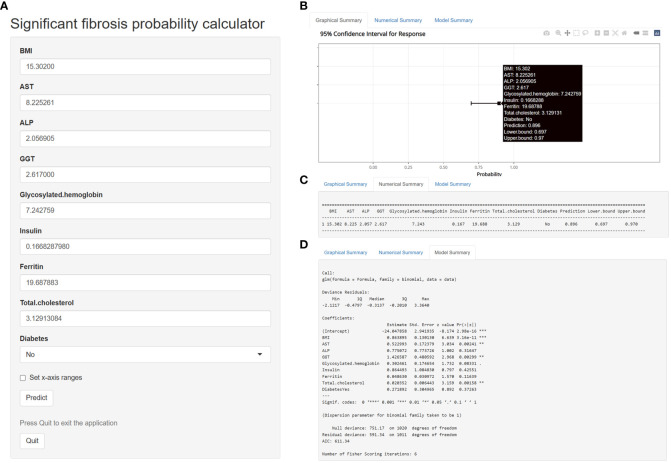
A calculator for predicting significant fibrosis probability in patients with nonalcoholic fatty liver disease.

Based on the multivariate logistic regression model constructed in this study, we generated a probability calculator for model visualization and easy operation (**Figure 7**). Enter the adjusted prediction variable at the corresponding position (**Figure 7A**) and click the ‘ Predict ‘ button to output the prediction result. The input predictors were adjusted as follows: BMI-> 10*log10 (BMI), AST-> exp [log10 (AST)], ALP-> log10 (ALP), GGT-> log10 (GGT), Insulin-> log10 [log10 (Insulin)], Ferritin-> exp [log10(Ferritin)]. Glycosylated hemoglobin-> 10*log10 (Glycosylated hemoglobin), Total cholesterol-> 1000*exp [- (Total cholesterol)].

For example, enter the following data in the web calculator, BMI=15.30200, AST=8.225261, ALP=2.056905, GGT=2.617000, Glycosylated hemoglobin=7.242759, Insulin=0.1668287980, Ferritin=19.687883, Total cholesterol=3.12913084, Diabetes=No. The probability and 95% CI of significant fibrosis in this patient can be obtained according to the prediction model we established. Based on the above data, the predicted probability was 89.6%, indicating that the patient was highly likely to have significant fibrosis (**Figures 7B, C**). **Figure 7D** shows the detailed parameters of this model. The application of this probability calculator greatly facilitates the diagnosis and treatment of liver fibrosis by clinicians. Anyone can use this tool at the following address: https://mydesign.shinyapps.io/significant_fibrosis_probability_calculator/?_ga=2.224708633.2084824833.1679065732-920179104.1679065732 (shinyapps.io).

**Figure 8:**
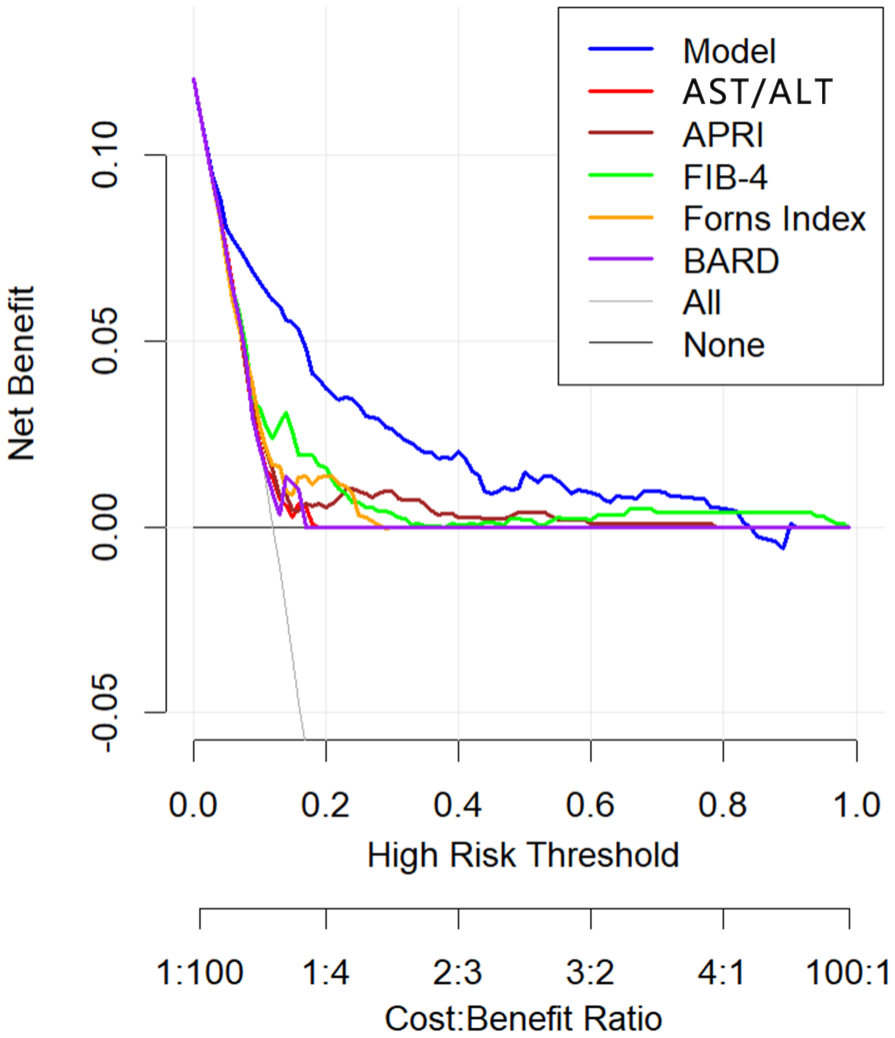
Decision curve analysis of non-invasive liver fibrosis prediction models. The x axis of the decision curve is the threshold probability, and the y axis is the net benefit. The threshold probability indicates that the expected benefit of treatment for patients diagnosed with significant fibrosis is equal to the point of loss from overtreatment. The x-axis at the bottom represents the ratio of loss to gain under each threshold condition.

DCA plots were plotted for six liver fibrosis prediction models, including this model (**Figure 8**). The DCA indicates that patient threshold probabilities in the range of approximately 0.1-0.8 add more net benefit to the use of this probability calculator than other diagnostic models when compared to strategies that treat all patients or no patients, indicating that the model is a good assessment tool.

## 4 Discussion

With the change of people ‘s lifestyle, the incidence of significant fibrosis in patients with NAFLD has become increasingly prominent and has attracted more and more researchers ‘ attention. Early diagnosis of liver fibrosis is helpful for the treatment of NAFLD patients. At present, the main diagnostic methods are serological examination and imaging examination. However, due to the immaturity of serological methods and the inability to accurately diagnose patients, they are generally used as diagnostic methods for primary medical care. The imaging method is limited by the site and professional technology and cannot be widely used. Therefore, there is still a lack of simple and accurate non-invasive methods to predict significant fibrosis in NAFLD patients. In this study, we developed a novel non-invasive model to predict the probability of significant fibrosis in patients with NAFLD, which consists of common biochemical indicators and clinical features. Compared with the previous scoring system, the new model has higher diagnostic accuracy.

A study of adolescent NAFLD patients showed that higher BMI can cause a greater risk of liver fibrosis in patients with severe hepatic steatosis (31) In this model, BMI level is positively correlated with the risk of liver fibrosis, that is, a high level of BMI will lead to an increase in the probability of liver fibrosis. Consistent with the above research results, it can be considered that BMI has a non-negligible effect on the occurrence of liver fibrosis. Diabetes is also a common disease in people with high BMI and is more common in people with chronic liver disease. Diabetic patients may develop fibrosis due to excessive production of adipose factor and lack of adiponectin, which stimulates collagen synthesis (32).As the model predicts, there is a positive correlation between diabetes and liver fibrosis. Compared with normal patients, patients with diabetes have a higher risk of liver fibrosis. In addition, studies (33, 34) have shown that serum AST is increased when liver injury occurs in patients with NAFLD, and it has been preliminarily confirmed that AST level is closely related to the occurrence of liver fibrosis in patients with NAFLD, which is highly similar to our findings. A previous study (35) showed that patients with advanced fibrosis (F3-F4) were older, more obese, more prone to diabetes and tened to have elevated levels of GGT compared to other groups, thus suggesting that GGT could be a biomarker for liver fibrosis in patients with NAFLD. The clinical application of ALP is relatively rare, but in a study of obese NAFLD patients, it was found that serum ALP can be used as an independent predictor of liver significant fibrosis in obese NAFLD patients, and through our model prediction results, ALP does have a strong positive correlation with liver fibrosis in NAFLD patients (36). As a necessary trace element, iron is stored in the liver in large quantities, and the occurrence of various diseases is related to the change of iron content. In recent years, A study (37) has reported that serum ferritin can successfully predict the development of liver fibrosis, Another study (38) has also shown that elevated serum ferritin in NAFLD patients does not imply the development of liver fibrosis in NAFLD patients, and in our experiment, a weaker positive correlation between ferritin (OR:1.02; 95%CI:1.01,1.03) and liver fibrosis was observed. Therefore, the relationship between ferritin and liver fibrosis remains to be discussed.

However, this study has some limitations. First, the data source of this study is relatively single, only including people from different regions of the United States for the survey and not from other different countries and regions, so the development of the model may have some geographical limitations. Secondly, due to the loss of follow-up or unqualified test of the subjects, there are more missing data in this study, which affects the effect of the model to a certain extent. Finally, due to the limitation of experimental equipment or professional and technical personnel, some clinical features or biochemical indicators have certain bias.

## 5 Conclusion

In summary, this study successfully constructed an excellent predictive model of liver fibrosis in NAFLD patients based on multivariate logistic regression analysis. This model can be used by frontline clinicians to predict liver fibrosis in patients with NAFLD, thereby reducing the need for unnecessary invasive liver tissue testing. In addition, we have developed a probability calculator based on this model to assist clinicians in making diagnoses for patients and to help them develop rational, individualized treatment plans for patients, greatly improving the diagnostic accuracy and treatment efficiency of liver fibrosis.

## Data availability statement

The raw data supporting the conclusions of this article will be made available by the authors, without undue reservation.

## Ethics statement

The studies involving human participants were reviewed and approved by NCHS Ethics Review Board. Written informed consent to participate in this study was provided by the participants’ legal guardian/next of kin.

## Author contributions

YG presents ideas, data collection, and processing. YG and BS data analysis, graphics, and manuscript writing. YL and YX project management, supervision, and review editing. All authors contributed to the article and approved the submitted version.
